# CultReal—A Rapid Development Platform for AR Cultural Spaces, with Fused Localization

**DOI:** 10.3390/s21196618

**Published:** 2021-10-05

**Authors:** Anca Morar, Maria-Anca Băluțoiu, Alin Moldoveanu, Florica Moldoveanu, Alex Butean

**Affiliations:** 1 Faculty of Automatic Control and Computers, University POLITEHNICA of Bucharest, RO-060042 Bucharest, Romania; maria_anca.balutoiu@upb.ro (M.-A.B.); alin.moldoveanu@cs.pub.ro (A.M.); florica.moldoveanu@cs.pub.ro (F.M.); 2 Faculty of Engineering, Lucian Blaga University of Sibiu, RO-550024 Sibiu, Romania; alex@butean.com; 3 S.C. INDUSTRIAL SOFTWARE S.R.L, RO-550173 Sibiu, Romania

**Keywords:** augmented reality, localization, beacons, computer vision

## Abstract

Virtual and augmented reality technologies have known an impressive market evolution due to their potential to provide immersive experiences. However, they still have significant difficulties to enable fully fledged, consumer-ready applications that can handle complex tasks such as multi-user collaboration or time-persistent experiences. In this context, CultReal is a rapid creation and deployment platform for augmented reality (AR), aiming to revitalize cultural spaces. The platform’s content management system stores a representation of the environment, together with a database of multimedia objects that can be associated with a location. The localization component fuses data from beacons and from video cameras, providing an accurate estimation of the position and orientation of the visitor’s smartphone. A mobile application running the localization component displays the augmented content, which is seamlessly integrated with the real world. The paper focuses on the series of steps required to compute the position and orientation of the user’s mobile device, providing a comprehensive evaluation with both virtual and real data. Pilot implementations of the system are also described in the paper, revealing the potential of the platform to enable rapid deployment in new cultural spaces. Offering these functionalities, CultReal will allow for the fast development of AR solutions in any location.

## 1. Introduction

Due to their pervasiveness and their capacity to display 3D content, smartphones have become very popular tools for augmented reality applications. Although other devices such as smart glasses are emerging [1], their price range and user interaction features still represent impediments to mass adoption. The AR domain has grown in popularity, as evidenced by the large number of AR platforms that offer a seamless integration of augmented content into the physical world. This perfect blending of the augmented and real worlds depends on the correct localization of the smartphone relative to the 3D coordinate system of the physical space. Even though many AR development platforms provide localization functionalities, it is still difficult to enable a time-persistent augmented reality experience, where all the visitors’ devices are localized relative to the same coordinate system, which is defined by a location manager. Such a platform is presented in this paper, which provides intuitive configuration and content definition functionalities covering multiple use cases, such as the rendering of 2D and 3D multimedia objects, guidelines, and quests. All these scenarios are enabled by the accurate, time-persistent localization of the visitor’s device. We used ARCore [2] technology, as well as a new localization method based on beacons and computer vision.

The paper is organized as follows. The second section presents a short overview of recent developments in the augmented reality field, with a focus on localization strategies and cultural experiences. The third section offers an overview of the CultReal architecture and a detailed description of the system, centered on our fused localization approach. Next, the evaluation of the system’s localization solution is presented. The fifth section describes pilot implementations in four cultural locations, covering various use cases. The final section presents several conclusions and discusses the potential of deploying the CultReal system in new cultural spaces.

## 2. Related Work

The augmented reality domain has grown in popularity, as evidenced by the huge number of commercial platforms [2,3,4,5,6,7,8,9] that provide 2D and 3D image recognition, tracking, position estimation, and the integration of augmented content to be overlaid on real scenes. Even though these platforms provide multiple and complex functionalities, there is no turnkey solution that covers all the use cases of augmenting a location with virtual content. Such a scenario is the localization of all the visitors in a common global coordinate system, defined by a location manager. Therefore, the augmented reality domain has also been explored by researchers who have adapted existing solutions or designed new ones depending on the various targeted use cases. This section briefly covers recent surveys and applications in the augmented reality field.

A very recent work [10] investigates the integration of the AR technology in different fields, such as education and culture, and analyzes future research directions. Another survey [11] also presents a historical overview of the augmented reality domain, illustrating the requirements and challenges of typical AR systems and identifying research opportunities in this area. These papers emphasize several challenges of the augmented reality domain that are relevant for our system. One of them is the bulkiness and high cost of specialized AR headsets, which represents an impediment for their wide adoption. An affordable alternative, also selected for the CultReal system, is the use of smartphones. Another challenge is the seamless integration of the virtual content within the physical world. The CultReal localization component provides an accurate estimation of the position and orientation of the user’s device, ensuring this seamless integration. Ling [12] analyzes academic contributions to the AR commercial domain and offers advice on how startups can take advantage of these developments when competing against senior companies. In [13], a survey on collaborative augmented reality is provided, identifying accomplishments, limitations, and future trends. The CultReal system addresses the multi-user challenge by rendering the same virtual content on multiple devices located in a common space. Grubert et al. [14] claim that most of the AR applications are used for one particular task and address the need for a continuous and multi-purpose user experience. They introduce a taxonomy for pervasive AR and context-aware AR, classifying the context, sources, and targets relevant for implementing a continuous AR experience. A very important purpose of the CultReal system is to provide such a continuous AR experience by ensuring the time-persistent localization of the users.

### 2.1. Localization in Augmented Reality Applications

Since one of the most important elements in the current work is the estimation of the position and orientation of the user’s device, this sub-section focuses on the localization problem in augmented reality applications.

The main algorithms of visual simultaneous localization and mapping (SLAM) [15], and their applications in augmented reality are investigated in [16]. The “AR Cross-center Interactive Device” solution [17] applies SLAM-based AR to interactive cultural exhibitions. Gao and Yang [18] introduce a 3D object recognition method in augmented reality applications using an improved Canny Edge detector, local feature descriptors, and the SLAM technology. Guido [19] is another AR system for indoor navigation, incorporating marker-based localization, SLAM, and inertial data. It uses the Unity’s AR Foundation Framework, a unified platform that includes core features from ARKit [3], ARCore, Magic Leap [9], and HoloLens [20]. An AR remote collaboration system with dense scene reconstruction [21] is used for remote guidance. This system generates a 3D mesh of the scene which can be explored and enriched with text and image annotations to provide instructions. Puigvert et al. [22] use the 3D feature points acquired by ARCore to obtain a sparse representation of a scene and provide a localization service based on the bags of binary words technique [23]. The time-persistence and collaboration requirements of AR systems, especially in complex industrial environments, are addressed in [24]. A map recovery and fusion strategy using visual-inertial SLAM have been proposed for the collaborative AR on multiple mobile devices. Ng et al. [25] use inertial sensors embedded in mobile devices and the IndoorAtlas technology [26], enabling indoor positioning and offering guidance to users through ARCore. A comprehensive survey on vision-based indoor localization, which also discusses the AR domain, is provided by Morar et al. [27]. An important component of the CultReal system is the ARCore localization technology, which fuses data from the smartphone camera and sensors though a visual-inertial SLAM. Recently, the combination of visual and inertial sensors has been successfully used for other high-granularity data processing environments, such as mobile X-ray detector tracking [28], indoor parking [29], and guidelines for indoor pedestrian navigation [30,31,32]. The validation of such approaches was possible due to a thorough analysis of synchronous multi-sensor datasets. We plan to construct a similar dataset for future versions of our work in order to verify the efficiency of our proposed localization.

An outdoor AR system that provides pose estimation of a mobile device relative to a 3D model of the environment, is proposed in [33]. The 3D model is reconstructed with the Structure from Motion technology [34], using images acquired by an unmanned aerial vehicle. Another outdoor AR system [35] applies position tracking based on a real-time kinematic global navigation satellite system (RTK GNSS) to register multiple maps from an existing SLAM system. A vehicle localization solution for AR-based assisted driving [36] uses odometry, camera recognized landmarks, and a map obtained with an extended Kalman Filter. Future versions of the CultReal system will also provide outdoor localization using GPS technology and other techniques identified in this paragraph.

Visual data can be fused with information from sensors to provide the accurate and fast localization of users or sensors in IoT environments. Baskaran et al. [37] introduce an AR interface for IoT devices using relational localization methods and metadata information acquired with the sensors. LESAR [38] is another localization system for environmental sensors which uses a smartphone camera with AR capabilities to measure the distances between sensors and the information provided by each sensor via Bluetooth signals. The current research uses beacons for an initial estimate of the user’s position and then provides accurate localization based on visual data.

### 2.2. Augmented Reality in the Cultural Domain

Even though augmented reality can be applied to various fields, the current research targets cultural spaces. Therefore, recent developments in the cultural domain are analyzed.

Intangible cultural heritage has recently become a popular topic [39] which comprises both inherited and living practices, which are inclusive, representative, and community-based. Zhao [40] proposes a digital protection method of intangible cultural heritage through AR, by fusing cultural digital content with realistic scene videos. The same problem is addressed in [41], where a natural user interface for engineers and scene reconstruction based on frames acquired with a mobile camera and through object tracking, are provided. GoFind! [42] is an AR system that allows the exploration of cultural heritage, providing location-based querying in historic multimedia collections, and an AR user interface that enables the overlay of photos onto the video stream of a mobile device. The CultReal system also provides an intuitive workflow to place virtual content into a cultural space and accurate localization functionalities to provide an immersive user experience.

A mobile AR teaching system that enables users to obtain information about historic buildings in the context of the Mackay culture is described in [43]. Silva and Teixeira [44] discuss the potential of developing an extended reality platform for immersive and interactive experiences, and propose new ways of exploring cultural heritage in the Serralves Museum and Coa Archeological Park. The “distant augmented reality concept” is introduced in [45], where a geo-based AR application for exploring cultural heritage in the Roman Baths of Ankara, using drones as video acquisition devices, is presented. A top-view rendering of the cultural site acquired by a drone, is displayed on the visitor’s mobile device. Alakhtar [46] provides an exploratory interview study regarding the use of AR in enhancing the tourism experience at heritage sites and proposes an intuitive user interface for exploring museum artifacts. A Microsoft HoloLens application for a small-scale exhibition at a café which provides an interactive experience on top of a collection of historic physical items demonstrates the usefulness of mixed reality [47]. Venigalla and Chimalakonda [48] describe the design and development of an augmented reality museum that provides relevant information and 3D views of artifacts. They also present a case study of the Online British Museum. The functionalities of the CultReal system are inspired from this analysis of existing AR solutions in the cultural domain. To cover a multitude of use cases, the system allows location managers to define various types of multimedia elements such as images, videos, or 3D objects, and to establish different event triggers (e.g., a video is played when the visitor approaches a beacon, a 3D object is displayed when the system detects a certain image in the real scene, a message is displayed after the user finishes the quest of identifying several items in the real scene). All these use cases are detailed in Section 5.

## 3. Proposed Method

**Figure 1:**
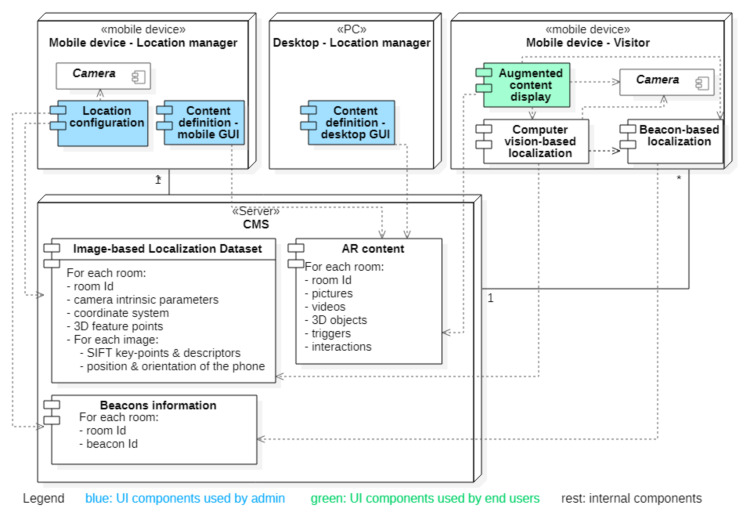
Deployment diagram of the CultReal System.

Figure 1 illustrates the architecture of the CultReal system as a server-based content management system (CMS) that stores multimedia content consisting of images, text, videos, and 3D objects. The multimedia content is defined by a location manager through the content definition component, which runs either on mobile or on desktop.

The proposed localization method requires a previous configuration of the space, which is accomplished by the location manager, following a simple workflow. The location configuration component produces image-based localization information for each room or area of interest in a cultural space. This information is stored in the CMS, along with information about the beacons that are placed in those spaces. The image based-localization data and the beacon information are further used by the computer vision-based localization component and the beacon-based localization component, which run on the visitor’s device. The augmented content display component renders the multimedia objects, superimposing them on the video flow acquired with the visitor’s smartphone camera based on the estimated position and orientation provided by the localization components. The computer vision-based localization service is intended for indoor use, but the beacons can be employed in both indoor and outdoor scenarios as long as their range capabilities are kept.

The accuracy of the ARCore localization technology was tested, considering HTC Vive [49] as a reference, obtaining promising results, as described in the Evaluation section and detailed in [50]. The main problem with this localization technology is the lack of a persistent global reference over time. The ARCore coordinate system is always set according to the position of the phone when starting the application (it practically resets when starting the application). This issue has been partially addressed in the ARCore SDK by introducing persistent cloud anchors [51]. Such an anchor is a 3D point in space which can be associated with a 3D object for viewing. When restarting the application using the same or another device, after a brief scan of the environment, that persistent anchor is positioned in the place where it was created. Thus, these anchors can be seen as persistent landmarks over time. The problem with these ARCore anchors is their short lifespan (24 h in older versions of ARCore, up to one year in newer versions). For this reason, they cannot be used in a system that aims to define multimedia content that is persistent over a very long period (several years). Also, the ARCore localization technology requires an initialization step of several seconds, when the user is tasked with performing scanning motions, to identify the most important planes in the scene and a set of feature points. This does not pose a problem for the location manager, but it would have a negative impact on the user experience of the visitors. In conclusion, the only solution was to implement a custom localization method which performs the initialization task for the ARCore technology, as detailed in Section 3.2.2. In a configuration step, the location manager defines a persistent coordinate system for each room or area of interest in the cultural space. The augmented content associated with a room is defined relative to the room’s coordinate system, as specified by the location manager. When a visitor enters a new room or area of interest in the cultural space, the beacon-based localization service identifies the closest beacon to the phone and downloads the image-based localization information associated with that beacon acquired in the configuration stage. In the computer vision-based initialization step, an image obtained from the visitor’s smartphone camera is matched with an image acquired in the configuration stage. Based on corresponding pairs of feature points in the two matched images, the position and orientation of the mobile device relative to the coordinate system of that room is estimated. The computed position and orientation information are then used as initialization data for the ARCore localization technology, acting as persistent cloud anchors. In this manner, the augmented content is always displayed relative to the same physical location, offering a time-persistent augmented reality experience.

The computer vision-based initialization method is based on the OpenCV library [52], which provides a number of functions that can be used in extracting feature points, mapping images, and estimating transformation matrices between images. We used the Perspective n-Point (PnP) algorithm [53] to compute the position and orientation of the visitor’s device relative to a coordinate system defined by a location manager. This algorithm requires a set of 3D points acquired in the configuration stage, and their matching 2D points detected in the localization stage, as further explained in Section 3.1 and Section 3.2.2.

**Figure 2:**
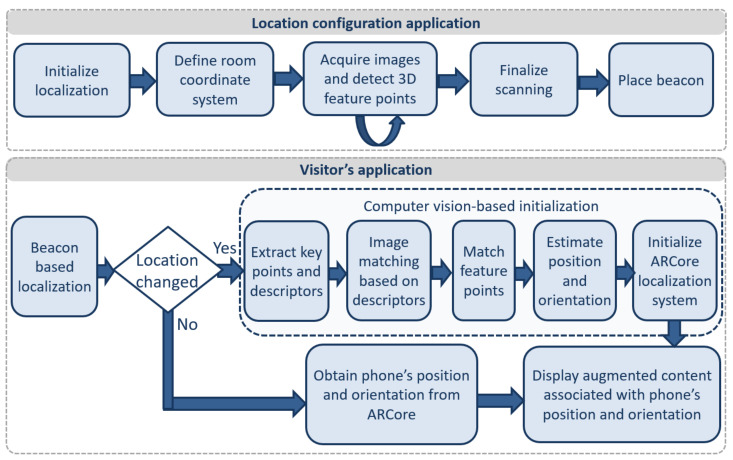
Workflows of the CultReal applications: location manager application (**top**) and visitor application (**bottom**).

The CultReal system contains two mobile applications. The first one is intended to be used by the location manager in the configuration stage. The second application runs on the visitor’s device, performs the localization task and displays the augmented content. The workflows of these applications are illustrated in Figure 2.

### 3.1. The Location Manager’s Application

In the configuration step, for each room or area of interest in a cultural building, the CMS is populated with localization information. The ARCore localization is applied to obtain the position and orientation of the location manager’s device. The ARCore technology uses simultaneous localization and mapping, as well as inertial data, to estimate the position and orientation of the phone’s camera relative to a coordinate system which is determined by the phone’s position when the application starts. To initialize the localization, the manager is required to navigate the space, making scanning motions to determine the most important planes of the room (floor, walls). For time-persistence and repeatability of the localization, regardless of the position of the phone when starting the application, the user (location manager) is asked to define a coordinate system for the room. The axes of this system are computed based on three user-defined points. The first point represents the origin of the coordinate system, while the second and the third points are used to determine the Ox and Oz axes, respectively. The application builds an orthogonal system whose xOz plane is superimposed on the floor.

Once the application has been initialized and a room coordinate system has been defined, the location manager moves the phone’s camera around the room and points it at all the walls and important elements, acquiring images of the space. During scanning, the AR technology detects 3D feature points which represent prominent points located, for example, at corners or in textured areas. The projections on these feature points on the acquired images will be matched against 2D key points in the computer-vision localization stage, as explained in Section 3.2.2. The algorithm for detecting 3D feature points using the ARCore technology is not public. Therefore, we tested several 2D feature detectors, including SIFT [54], SURF [55], and ORB [56], and observed that a relatively large percentage of points obtained by projecting the ARCore 3D feature points (approximately 50%) had similar coordinates with points produced with the SIFT algorithm. In comparison, the ORB and SURF algorithms led to considerable smaller percentages (17 and 31%, respectively) of common feature points. Therefore, for a consistent localization between the configuration and the localization stages, the SIFT key points and descriptors for each acquired image are also stored in the CMS.

During configuration, the beacons are also placed in the rooms or areas of interest, and their IDs are stored in the CMS along with the image-based localization information.

**Figure 3:**
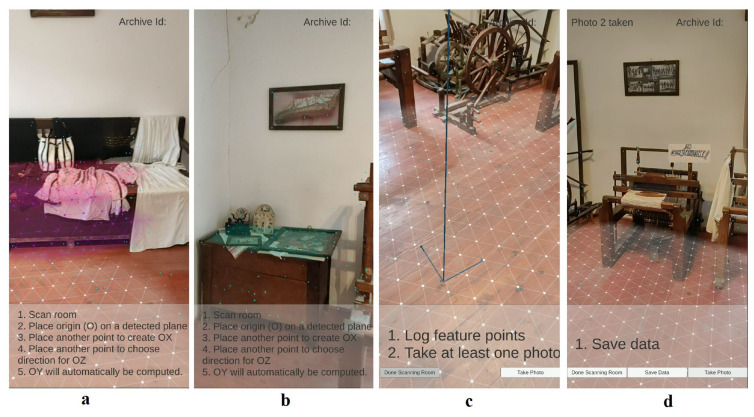
Workflow of the location manager application displaying the guidelines and several prominent planes: the floor and several planar surfaces of a bed (**a**), displaying detected feature points (**b**), displaying the user-defined coordinate system (**c**), and displaying a message that a photo has been taken (**d**).

The steps of the location configuration workflow (with screenshots from a phone running the configuration application at the Badea Cârțan Museum [57], in Figure 3) are summarized below:

1. The ARCore technology is initialized while the location manager performs scanning motions. In this step, ARCore identifies a series of important planes, such as the floor and the walls. The intrinsic parameters of the user’s phone are stored in the CMS.
2. A room coordinate system is computed based on three user-defined points located on the floor plane.
3. During the scanning, 3D feature points are determined by ARCore. The location manager acquires images of the space. For each image, the position and orientation of the user’s phone, as determined by ARCore, are saved in the CMS.
4. When the scanning is finalized, each image is processed with the SIFT algorithm to produce key points and descriptors, which are saved in the CMS.
5. A beacon is placed in the room/area of interest, and its ID is stored in the CMS.

### 3.2. The Visitor’s Application

The visitor’s application runs two localization services, one that computes an approximate location based on beacons, and one that employs computer vision to accurately estimate the position and orientation of the visitor’s phone. When the visitor changes rooms, the new room’s beacon is identified, the CMS content associated with the beacon is downloaded, and the computer vision-based localization initialization step is performed. The aim of this step is to initialize the ARCore localization system with the origin of the coordinate system associated with the room. After that, the computer vision-based localization component can use the phone’s position and orientation given by ARCore. The workflow of this application, illustrated in the bottom image of Figure 2, is summarized below:

1. The beacon-based localization component identifies the beacon which is closest to the visitor’s phone. This approximate localization greatly reduces the search space of the computer vision component. Only the images associated with the position of the current beacon are sent from the CMS to the computer vision component.
2. In the computer-vision based initialization step, the SIFT feature points and descriptors from the current image acquired with the visitor’s phone are extracted. Then, the image processing module matches the current frame of the phone’s video camera with the most similar image from the CMS based on the corresponding feature points and descriptors in the two images. Using the information associated with the matching image, the current position and orientation of the visitor’s phone are estimated. This information is then used to initialize the ARCore localization.
3. The multimedia content is displayed, superimposed naturally over the video stream of the phone, and associated with the phone’s position and orientation.

#### 3.2.1. Beacon-Based Localization

**Figure 4:**
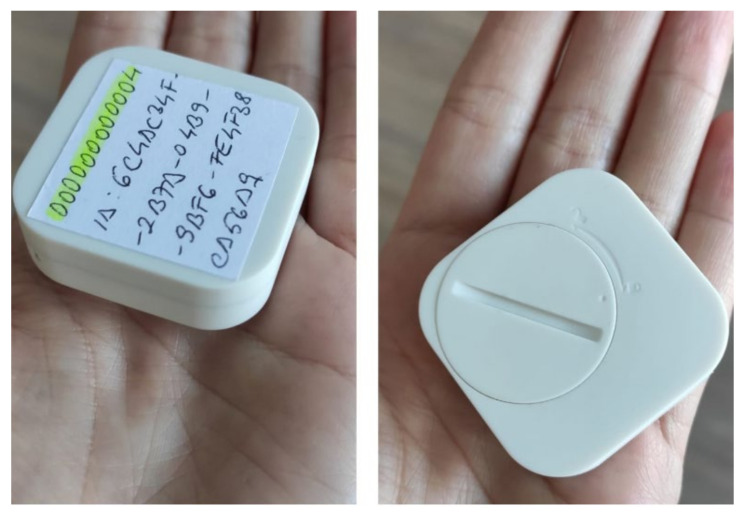
iB004N beacons used for beacon-based localization.

The beacon-based localization component uses an Android library that returns a list of nearby beacons. Within the CultReal system, iB004N beacons (Figure 4) were used, configured to send information via the Eddystone protocol [58]. Beacons are identified by a *namespaceId* and an *instanceId*. In practice, the *namespaceId* is the same for all sensors—so that the beacons can be differentiated from those exposed by other entities. The *instanceId* is unique for each beacon. Other settings that can be made are related to broadcast power, range, broadcast interval and received signal strength.

The beacon-based localization library scans, at certain time intervals, the signals sent by the nearby beacons and returns a list of those detected, in ascending order of distance from the smartphone. The core of the library is a service, an Android component that runs in the background. The beacon-based localization library exposes two main methods: *startService*() and *stopService*(). The result of the scan is sent to the computer vision component using the broadcast mechanism through which messages are sent or received from the operating system or between Android applications.

The beacon-based localization component is implemented using the Android Beacon Library [59], version 2.16.2. The library’s application programming interface (API) provides approximate information about the distance to the beacons (in meters) based on the power of the Bluetooth signal detected by the smartphone. These values are influenced by other factors and may vary at different times. The purpose of the application is to detect the nearest beacon and not the exact distance to it. At the end of each scan, the component outputs a list of beacons identified by *namespaceId*, *instanceId* and *distance*. In addition to this information, image-based localization data regarding the room in which each beacon is located is also required. This data, stored in the CultReal CMS, is exposed through a web service that the library calls at a given time, and then is saved locally on the visitor’s mobile device to avoid multiple calls. For a reliable end result, a mechanism is implemented that keeps a history of the closest beacons scanned in the last seconds and the distance to them. If the result of a scan returned by the Android Beacon Library is null or does not contain the beacons that were in very close proximity the previous seconds (not even at a great distance), the local history is used.

#### 3.2.2. Computer Vision-Based Initialization Step

In the initialization step, the computer vision-based localization component matches an image acquired with the visitor’s phone against the most similar image in the CMS. It also computes the corresponding feature points between the matched images and applies the PnP algorithm to estimate the position and orientation of the visitor’s device relative to the coordinate system defined by the location manager.

As previously mentioned, in the location configuration stage, 3D ARCore feature points are stored in the CMS. For all the images acquired by the location manager, SIFT key points and descriptors are also saved in the CMS. In the actual initialization stage, the SIFT detector is applied on the current frame acquired with the visitor’s phone, producing SIFT key points and descriptors.

The image mapping algorithm determines the image in the CMS that most closely resembles the image corresponding to the current frame acquired from the visitor’s phone, based on the previously computed descriptors. For each image in the CMS, the mapping algorithm identifies the two best matches for each descriptor from the visitor’s image, with a k-nearest neighbor strategy and the fast library for approximate nearest neighbors (FLANN) descriptor matcher [60]. We performed a Lowe’s test [61] on these matches, using a ratio threshold of 0.75. This value was chosen as to find a balance between eliminating false matches while not discarding correct matches, following the guidelines from the OpenCV documentation and from Lowe’s work. The image from the CMS with the most passed tests represents the match for the image acquired with the visitor’s phone.

**Figure 5:**
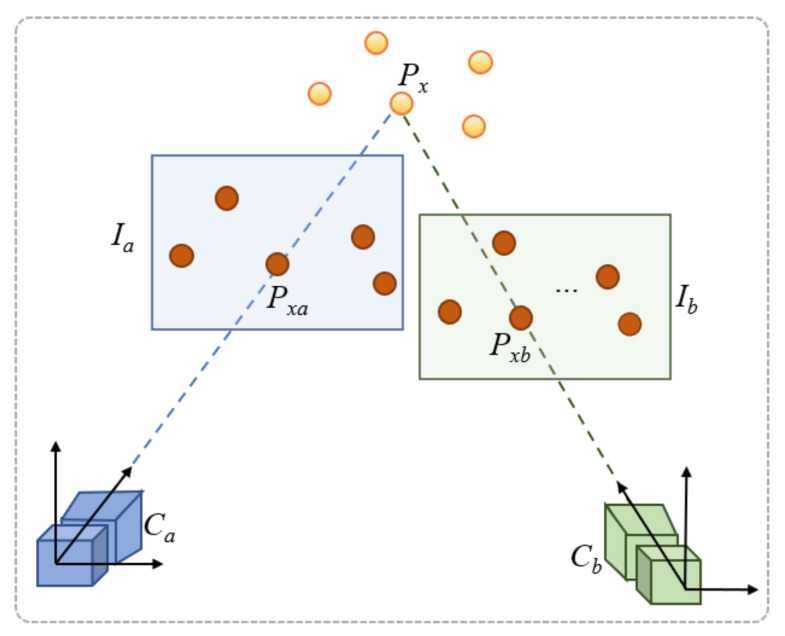
Estimation of transformation matrices between images *I_b_* and *I_a_*.

Next, the PnP algorithm [53] estimates the position and orientation of the visitor’s camera relative to the coordinate system of the image from the CMS that most closely resembles the image acquired with the visitor’s phone. The PnP algorithm calculates a transformation matrix for the video camera *C_b_* of an image *I_b_* (Figure 5) relative to the coordinate system of the video camera *C_a_* of another image *I_a_*, if the following elements are known:

- The intrinsic parameters of the two cameras, *C_a_* and *C_b_*.
- A series of 3D points in the coordinate system of camera *C_a_*.
- Aheir corresponding 2D points in image *I_b_*.

Thus, after identifying in the CMS the image (*I_a_*) that most closely resembles the image acquired with the visitor’s phone (*I_b_*), 3D points corresponding to image *I_a_* and the matching 2D feature points corresponding to image *I_b_* need to be identified. When running the PnP algorithm, only the feature points that are detected both by ARCore and SIFT are considered. Therefore, after identifying the image from the CMS that has the most similarities with the visitor’s image, the set of SIFT key points is filtered. Only the points that correspond to the feature points that were detected both by ARCore and SIFT are selected. To identify these correspondences, the ARCore 3D feature points are projected on the image from the CMS, producing 2D feature points (henceforth, ARCore 2D feature points).

The projections are computed based on the 3D coordinates of the points, the position and orientation of the phone’s camera for the image, and the intrinsic parameters of the camera. A maximum distance threshold of 10 pixels in the image space is applied to consider a match between a 2D ARCore feature point and a SIFT key point (SIFT plus ARCore).

**Figure 6:**
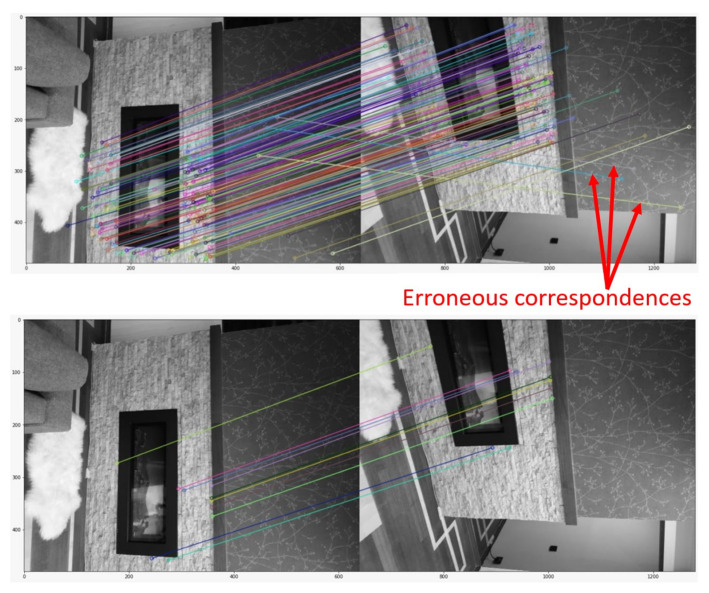
Correspondences detected with matching algorithms: results of the FLANN algorithm (**top**) and of the BFMatcher (**bottom**).

The PnP localization algorithm is sensitive to correspondence errors. For this reason, it is very important that the pairs of matching feature points in the two images, *I_a_* and *I_b_*, are detected correctly. We experimented with two matching algorithms provided by OpenCV, FLANN [62], and brute force matcher (BFMatcher) [63]. These algorithms were compared regarding accuracy and computing time, as detailed in Section 4.3. The BFMatcher detects fewer pairs of feature points, but also fewer erroneous correspondences, as illustrated in Figure 6. Even though FLANN algorithm is considerably faster than BFMatcher, the accuracy of the localization represents a more important metric. Therefore, the BFMatcher algorithm was selected, and to further increase the accuracy of the localization method, the results were filtered as explained below. Running the BFMatcher on two images, a set of corresponding feature points is obtained. For the two images placed side by side (as in Figure 6), for each pair of corresponding feature points, the direction of the vector connecting the two feature points is computed in image space. We also determined the average direction of the vectors connecting pairs of feature points and we eliminated the outliers, i.e., the pairs whose direction differed significantly from the average direction. A threshold of approximately 30 degrees was used for the direction error, corresponding to a cosine of 0.85, to identify the outliers.

The PnP algorithm can determine the position and orientation of the visitor’s camera (*C_b_*) relative to the coordinate system of the video camera (*C_a_*) used to acquire the image in the CMS. Since all images in the CMS are labeled with position and orientation data relative to the coordinate system of the current room or area (as defined by the location manager in the configuration stage), the position and orientation of the visitor’s camera in that coordinate system can also be determined.

The steps of the computer vision-based initialization workflow, when the visitor enters a new room, are summarized below:

1. An image *I_b_* is acquired with the visitor’s smartphone camera.
2. The SIFT detector is applied on image *I_b_*, identifying a set of key points, *P_b_SIFT_*, and a set of descriptors, *D_b_SIFT_*.
3. The image matching step identifies, in the CMS, the most similar image to *I_b_*, namely *I_a_*, by running the OpenCV FLANN-based descriptor matcher with a k-nearest neighbor strategy on the descriptors from *D_b_SIFT_* and all the sets of descriptors from the images in the CMS.
4. The set of 3D feature points, *P_ARCore_*, extracted with ARCore in the configuration stage, is retrieved from the CMS. Next, these 3D points are projected on image *I_a_*, producing 2D ARCore feature points. For the next steps, only the 2D feature points from *P_a_SIFT_*, corresponding to the projections of the 3D points from *P_ARCore_* on image *I_a_*, are considered (producing the set *P_a_SIFT+ARCORE_*).
5. The BFMatcher is run, to determine the set of feature points from *I_a_*, *P_a_SIFT+ARCORE_* corresponding to the feature points from *P_b_SIFT_*. We filter the results and remove the outliers, i.e., the matching pairs whose direction deviates from the average direction. The best 20 matches are selected for the PnP algorithm. The final matched features are stored in the following sets: the set of final feature points in image *I_b_*, *P_b_* = {*P_xb_*}, the set of equivalent feature points in image *I_a_*, *P_a_* = {*P_xa_*}, and the set of 3D corresponding feature points, *P* = {*P_x_*} (as illustrated in Figure 5).
6. The PnP algorithm is run with the following parameters: the intrinsic parameters of the cameras *C_a_* and *C_b_*, the set of selected 3D feature points *P_x_* in the coordinate system of camera *C_a_*, and their corresponding 2D feature points *P_xb_*, in image *I_b_*. The PnP algorithm outputs the transformation matrices of camera *C_b_* relative to the coordinate system of camera *C_a_*.
7. The position and orientation of camera *C_b_* are computed in the coordinate system of the current room (defined by the location manager in the configuration stage). This information is used as initialization data for ARCore localization.

The Evaluation section presents the accuracy of the PnP algorithm, with either FLANN or BFMatcher in the image matching step. Also, computing times are given for various tasks of the localization, with or without the proposed filtering method in the image matching step.

The proposed computer vision-based initialization algorithm can be time consuming, especially on medium-level smartphones. For quick response in the visitor’s application, we exploit the speed and accuracy of the ARCore localization technology. Therefore, the proposed fused localization is applied only once, when the visitor enters a new room. The estimated position and orientation of the visitor’s smartphone, computed with the PnP algorithm, acts like a persistent cloud anchor, being used as initialization data for the ARCore localization technology. After this initialization step, the ARCore technology performs the localization task, but relative to the room’s coordinate system defined by the location manager, and not relative to the default ARCore coordinate system. The proposed computer-vision based localization runs on a separate thread, without blocking the video stream of the smartphone’s camera.

The computer-vision localization component is very useful when placing multimedia content bound to the 3D coordinate system of a certain location, but not necessarily to a real object in that location. Another use case, when a virtual object is, for example, bound to a painting or a poster from the real world, can be better handled with the Augmented Images API [64] provided by ARCore, which stores 2D features of an image provided by a location manager, and, at runtime, searches for these features on flat surfaces in the user’s environment. When identifying the features, it displays the augmented content that is bound to that image. This functionality was also integrated in the CultReal framework, to cover all possible use cases when deploying the solution in a new cultural setting.

#### 3.2.3. Augmented Content Display

The augmented content display component renders the multimedia objects in the 3D coordinate system defined by the location manager and places the virtual camera according to the estimated position and orientation of the visitor’s smartphone camera. Thus, the 3D content is integrated seamlessly in the real world, being displayed over the video stream provided by the visitor’s phone camera. Besides 3D models, texts, images, and videos positioned relative to a room’s 3D coordinate system, the module can also render 2D text and videos in the image space.

## 4. Evaluation

Several evaluation tasks were performed to assess the accuracy of the ARCore localization technology and that of the proposed localization solution.

### 4.1. Beacon-Based Localization

**Figure 7:**
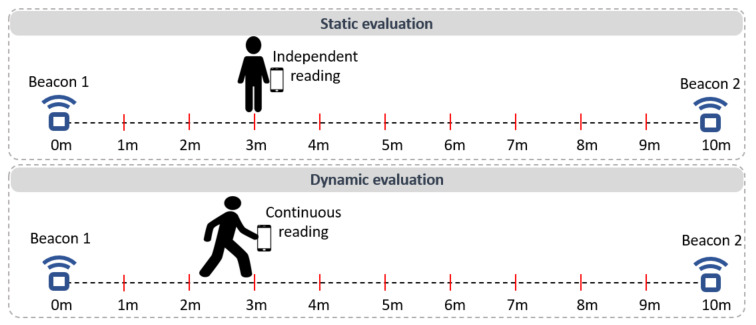
Evaluation of the beacon-based localization: static scenario (**top**) and dynamic scenario (**bottom**).

The accuracy of beacon-based localization was evaluated in two scenarios: static and dynamic one. We used a measuring tape to establish the positions of two beacons located 10 m apart. Also, on the distance between the two beacons, checkpoints were defined every 1 m, as illustrated in Figure 7.

In the static scenario, a mobile device running the beacon-based localization service was placed by a user at each checkpoint, and the closest beacon identified by the localization component was recorded. To ensure independent readings, the application was turned off and on again at each checkpoint. The application behaved as expected for the 0, 1, 2, 3, 7, 8, 9 and 10 m checkpoints: for the checkpoints in the 0–3 m interval, the closest identified beacon was Beacon 1, while for the checkpoints in the 7–10 m interval, the closest identified beacon was Beacon 2. However, for the checkpoints 4, 5, and 6 m, the readings oscillated between the two beacons.

In the dynamic scenario, the user holding the mobile device walked at different speeds from Beacon 1 to Beacon 2, as depicted in Figure 7 (bottom). Continuously reading the output of the localization service, we identified the distance from Beacon 1 when the output representing the closest identified beacon was switched from Beacon 1 to Beacon 2. For a slow user speed (0.17 m/s), the switch happened at 4.82 m distance from Beacon 1. For a normal user speed (0.23 m/s), the switch happened at 5 m, and for a fast user speed (0.33 m/s), the switch happened 6.48 m from Beacon 1.

These experiments reveal that the readings of the beacon-based localization service have a relatively large localization error (several meters) and are influenced by the speed of the user, due to delays in the Bluetooth communication. However, the drawbacks of beacon-based localization do not pose a problem for the current solution, since this technology is used only to obtain an approximate position of the user’s device.

### 4.2. ARCore Localization Technology Compared with HTC Vive

The HTC Vive system is very popular among virtual reality systems, partially due to its tracking technology which provides accurate localization of the head mounted display (HMD) device and of other components such as controllers and trackers [65,66,67]. HTC Vive was used as a reference system for assessing the accuracy of the ARCore localization technology. We paired a smartphone running an ARCore application and an HTC Vive tracker. The evaluation system consists of four applications:

- An HTC Vive management application which sends data about the position and orientation of the Vive tracker, as well as timestamp information, to a central application.
- An ARCore management application which sends data regarding the position and orientation of the smartphone (estimated with the ARCore technology), as well as timestamp information, to a central application.
- A Unity central application which receives position and orientation data for the Vive tracker and for the smartphone, and displays them in a common coordinate system
- An evaluation application that displays graphics with the trajectories and orientations provided by the HTC Vive and the ARCore applications, allowing the visualization of the position and orientation errors of the ARCore localization technology, relative to the HTC reference system.

The ARCore localization was tested on a Samsung Galaxy S7 Edge and on a Samsung Galaxy S9 Plus, on recordings ranging from 16 s to a couple of minutes. Overall, we obtained an average position error of 0.16 m and an average orientation error of 7.92 degrees, demonstrating the high accuracy of the ARCore localization technology for small-scale settings. More details about the experiments and the test results are presented in [50].

### 4.3. The Proposed Computer Vision-Based Localization in Virtual Environments

The image processing algorithms described in Section 3.2.2, i.e., the key points and descriptors extraction with SIFT, the image matching, the feature matching, and the estimation of the position and orientation with PnP, can introduce errors when integrated and working with real data. Thus, we decided to evaluate the localization system in stages, using synthetic data.

One possible identified problem was the accuracy of the OpenCV PnP algorithm, as well as the provided input, i.e., the intrinsic parameters of the cameras, the 3D feature points, and their projections on the images. To test the proposed localization algorithm, we created a virtual environment in Unity that contained three video cameras and a series of 3D feature points. The first virtual video camera, *VC_a_*, is the reference camera, corresponding to the position and orientation from an annotated image in the CMS, in the real system. The second video camera, *VC_b_gt_*, represents the ground truth camera, corresponding to the actual position and orientation of the visitor’s device in the real system. The third video camera, *VC_b_*, represents the camera for which we compute the transformation relative to *VC_a_*. This camera corresponds to the estimated position and orientation of the visitor’s device in the real system, computed with the PnP algorithm. Through a user interface, the positions and orientations of the *VC_a_* and *VC_b_gt_* video cameras, as well as the positions of the 3D feature points in the scene, can be modified.

For the *VC_a_* camera, the positions of the 3D feature points are given. The 3D points are projected on the image corresponding to *VC_b_gt_*, based on the intrinsic parameters of that camera, obtaining 2D feature points. The same 2D feature points are considered for the *VC_b_* video camera. The PnP algorithm computes a transformation matrix of a camera, *VC_b_*, relative to another camera, *VC_a_*, based on the 3D points associated with *VC_a_* and the corresponding projected 2D feature points associated with *VC_b_*. The differences between the positions and orientations of cameras *VC_b_* and *VC_b_gt_* represent the estimation error of our localization algorithm.

Table 1 shows the position and orientation of camera *VC_b_gt_* (ground truth) and the position and orientation of camera *VC_b_* (the one calculated using the PnP algorithm) for five configurations, as well as the localization error. The position error was computed as the distance between the position of camera *VC_b_gt_* and the position of camera *VC_b_*. The orientation error was computed as the angle (in degrees) between the orientation of camera *VC_b_gt_* and the orientation of camera *VC_b_*, using Unity’s *Quaternion.Angle* function.

Overall, an average position error of 0.0314 m and an average orientation error of 0.0551 degrees were obtained. Even though these results are very promising, they reflect only partially the efficiency of the localization method, since the evaluation was performed on virtual data. To further assess the accuracy of the proposed method, the localization application was run on real data.

### 4.4. The Proposed Computer Vision-Based Localization on Real Images

To test the proposed localization algorithm on real data, the ARCore localization technology was chosen as reference, providing the ground truth information. The steps of the evaluation methodology are presented below:

1. The location configuration application (described in Section 3.1) is run, and an image *RI_a_* is acquired. The position and orientation of the camera, given by ARCore, are saved.
2. An image *RI_b_* is then acquired, for which the position and orientation, computed with the ARCore localization technology, are also saved (representing the ground truth).
3. The room is scanned to determine ARCore 3D feature points.
4. After completing the scan, the 3D feature points are projected on image *RI_a_*, obtaining a series of 2D ARCore feature points.
5. The SIFT detector is run on image *RI_a_*, obtaining a set of SIFT key points and descriptors. In the image matching step, we use all the SIFT descriptors extracted from image *RI_a_*. For the PnP algorithm, we use only those points that are detected both by ARCore (points that correspond to projections of the ARCore 3D feature points) and by SIFT.
6. The SIFT detector is run on image *RI_b_*, obtaining a series of 2D feature points.
7. The image matching step (FLANN or BFMatcher) identifies pairs of 2D feature points from images *RI_a_* and *RI_b_*.
8. The PnP algorithm computes the position and orientation of the video camera corresponding to image *RI_b_*, based on the known position and orientation of *RI_a_*, the 3D feature points associated with image *RI_a_*, and the corresponding 2D feature points associated to *RI_b_*.
9. The results of the PnP algorithm are compared with the ground truth provided by ARCore.

The PnP-based localization algorithm was tested on a series of real images according to the sequence of steps described above. While Table 2 presents the position and orientation accuracy, Table 3 illustrates the running times for various steps of the computer vision-based localization method, on the same images. Table 2 shows the positions and orientations computed with the PnP algorithm compared to the ground truth, determined with the ARCore localization technology. This table also presents a comparison between the results of the localization, using different feature matching algorithms, i.e., FLANN and BFMatcher. For the BFMatcher we show the results of the localization with and without our proposed filtering.

Overall, the localization method based on the FLANN feature matcher obtained a position error of 0.573 m and an orientation error of 29.561 degrees. Both methods based on BFMatcher obtained similar orientation errors (approx. 15 degrees), but the filtering of the BFMatcher reduced the position error by 0.032 m. It should be noted that even though FLANN produces relatively accurate results, it is not as stable as BFMatcher. This can be observed in Table 2, entry 4, where the results of the PnP algorithm with input from the FLANN matcher deviate greatly from the ground truth.

Table 3 presents the computing time of the SIFT detector, the number of detected SIFT key points, the computing times of the feature matching algorithms (FLANN, BFMatcher with and without the proposed filtering), and the final number of matched feature points, used as input for the PnP algorithm. We also provide the running time of PnP. All the tests were made on a Samsung S9 Edge smartphone. Additionally, we include the computing time for filtering the 2D feature points, selecting only those that are detected both by SIFT and by ARCore, and the number of SIFT plus ARCore points. It must be pointed out that this step can be performed either in the localization stage, only for the image from the CMS that most closely resembles the visitor’s image, or in the configuration stage, but for all the images from the CMS acquired for a certain room.

As previously mentioned, the FLANN matching algorithm is faster than the variations of the BFMatcher, as can also be observed in Table 3. Comparing the average computing times for the FLANN based approach and for the feature matching using BFMatcher and filtering, we obtained a difference of approximately 188 ms. However, considering the importance of the accuracy in estimating the position and orientation of the visitor’s smartphone, we decided to use BFMatcher with the proposed filtering in the feature matching step. On average, the chosen computer vision-based initialization step runs in approximately 1.4 s if the selection of ARCore plus SIFT feature points are performed in the localization stage, and in approximately 0.76 s if this selection is performed in the configuration stage. It should be emphasized again that this computer-vision based localization step is applied only once, as an initialization step, when the visitor enters a new room. Then, the ARCore localization technology can be used. Therefore, the overall computing time of the initialization step does not represent an impediment.

## 5. Pilot Study

The CultReal solution was deployed in four cultural settings: the Museum of the University POLITEHNICA of Bucharest (UPB) [68], the UPB Library [69], the Badea Cârțan Museum [57], and the Gong Theatre [70]. We tested multiple scenarios to assess the potential of deploying the system in any new cultural location and to cover as many use cases as possible, as shown in the Supplementary Materials.

### 5.1. Beacon-Based Localization

A simple use case is to detect the room (or section of a hall) where the visitor is positioned and to render general information about that room. This use case is covered by the beacon-based localization component. Several beacons are placed in the cultural space, one beacon in each room/area of interest. When the beacon-based localization component detects the approximate position of the visitor’s smartphone, the application downloads on the mobile the general content related to that room or section. Then, the augmented content display component renders the content, which consists of a text or a video, in image space, without considering the actual position and orientation of the phone relative to the 3D room’s coordinate system, but only the presence of the phone in that room.

**Figure 8:**
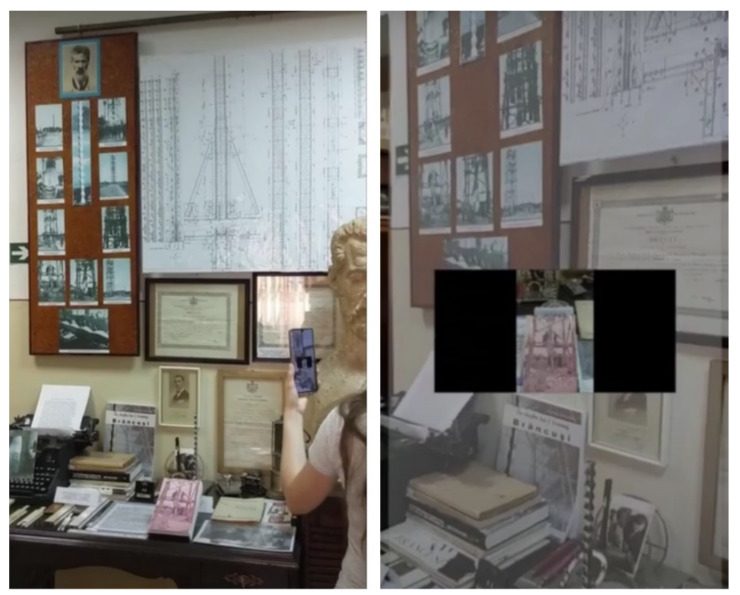
Piloting the beacon-based localization: a screenshot from an external video, filming the real scene and the visitor’s phone (**left**) and a screen capture of the phone, displaying a video with general information about that room, in the center, superimposed on the video stream from the phone’s camera (**right**).

This use case was tested both indoors and outdoors. Figure 8 illustrates such a scenario, where a beacon was placed in a section of the UPB Museum, dedicated to the Endless Column [71], which was designed by the Romanian sculptor Constantin Brâncuși. When the visitor is nearing that beacon, a video with general information about the column is displayed on the phone’s screen.

### 5.2. Augmented Images Functionality

The Augmented Images API provided by ARCore searches, in the real world, for images placed on flat surfaces and displays augmented content that is bound to those images. We designed several scenarios to test this functionality. Augmented images can be used for quests, to display guiding cues, or to simply render augmented content related to an image placed on a flat surface.

Quests are simple, yet entertaining elements that provide a rich user experience for the visitors of a cultural space. A quest was designed where the visitor was tasked to find several photos/paintings of personalities or other important items from a museum. When the phone application identifies in the real world a photo or a painting from the list, it displays a text announcing which of the tasks is complete. When all the items have been found, another text is displayed, informing the visitor about the successful completion of the quest.

Guiding cues are also simple, yet very useful features in an augmented cultural space. One possible scenario is to bind these guiding cues to pictures that represent logos or descriptions of locations of interest from the real world. Such a scenario was implemented in the grand hall at the UPB Museum, where guiding cues were displayed as arrows pointing to various sections of the hall, each section representing a faculty from UPB. For each faculty logo that was identified by the Augmented Images API, two arrows pointing at the nearby faculty booths were displayed on the visitor’s phone screen.

**Figure 9:**
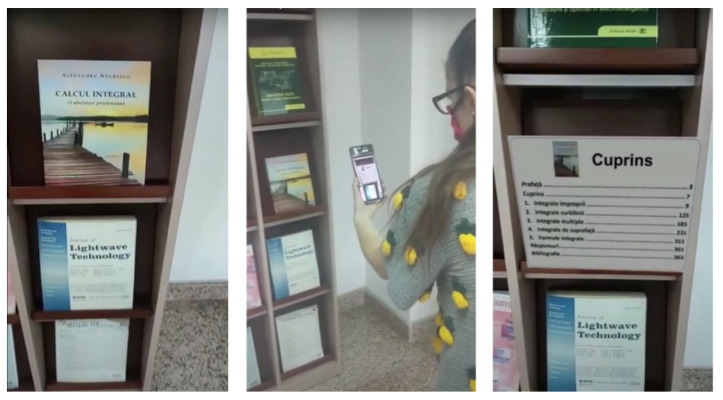
Piloting the augmented images functionality: a screen capture of the visitor’s phone, displaying the video stream acquired with the camera before identifying the book cover (**left**), a screenshot from an external video filming the real scene, and the visitor’s phone (**middle**), and a screen capture of the phone displaying the virtual image with the book’s table of contents superimposed on the video stream from the phone’s camera (**right**).

As previously mentioned, the augmented images functionality can be also used to display general multimedia content that is bound to a specific picture, poster, or painting. Several scenarios were tested, in which videos, 3D objects or images were displayed when identifying posters or book covers. Figure 9 illustrates a scenario where the application displays the table of contents for a book at the UPB Library.

**Figure 10:**
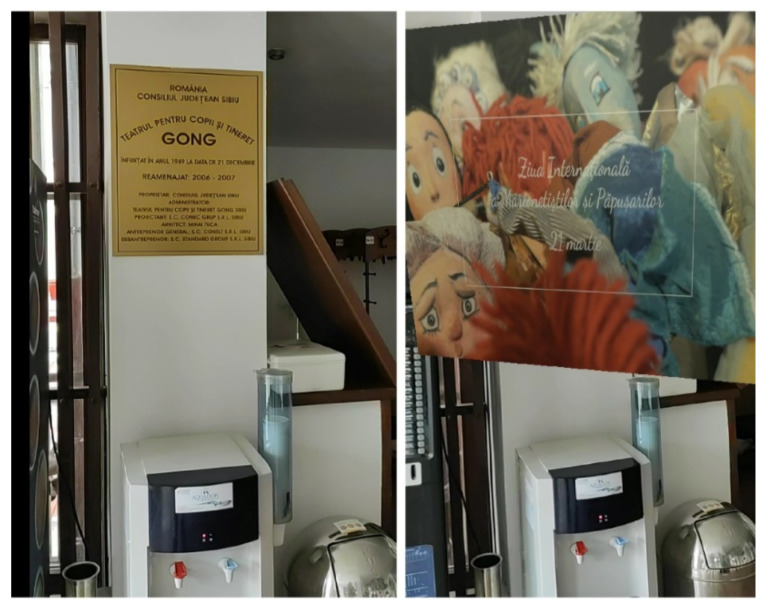
Piloting the augmented images functionality: a screen capture of the visitor’s phone, displaying the video stream acquired with the camera, before identifying the logo of the Gong Theatre (**left**), and a screen capture of the phone displaying the video promoting a cultural event superimposed on the video stream from the phone’s camera (**right**).

Figure 10 showcases a scenario where the application displays a video promoting a cultural event at the Gong Theatre, connected to the physical logo of the theater.

### 5.3. The Computer Vision-Based Localization

The computer vision-based localization component estimates the position and orientation of the visitor’s phone relative to a room’s coordinate system, defined by the location manager. If multimedia content is placed relative to that coordinate system, then the augmented display component renders this content, then superimposes it on the video stream acquired with the visitor’s smartphone camera. Several scenarios were tested, populating the CMS with content associated with rooms or areas of interest from the cultural spaces. Each 3D image, text, video, or mesh stored in the CMS was assigned to a specific area and placed relative to the coordinate system defined for that area, based on a transformation matrix containing translation, rotation, and scaling.

**Figure 11:**
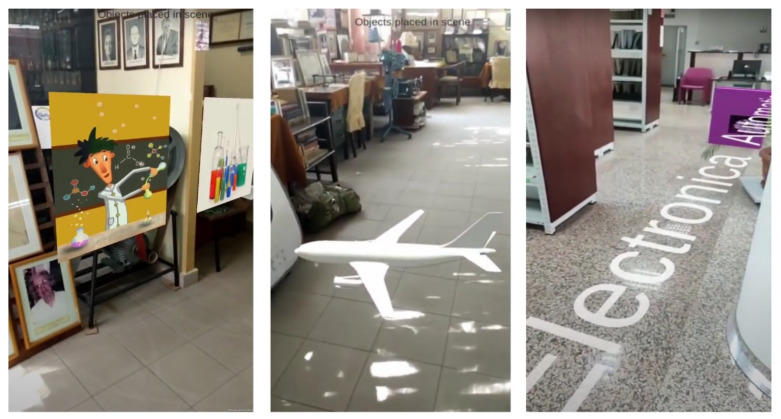
Piloting the computer vision-based localization: screen captures of the visitor’s phone, displaying two virtual images (**left**), a 3D model of a plane (**middle**), 3D text placed on the floor (**right**).

Figure 11 illustrates three of these scenarios. The image on the left shows a screen capture of the visitor’s phone displaying two virtual images of chemistry experiments, placed in the 3D coordinate system associated with a section of the UPB museum. In the middle, a 3D model of a plane is assigned to another section of the UPB museum. The image on the right shows a 3D text superimposed on the video stream acquired with the visitor’s phone camera, at the UPB library.

**Figure 12:**
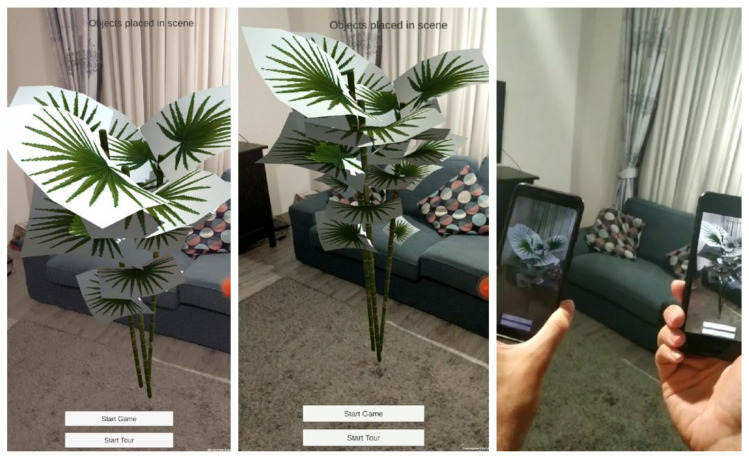
Piloting the computer vision-based localization with multiple users in a common augmented space: a screen capture of the first phone, displaying a 3D model of a bamboo (**left**), a screen capture of the second phone, displaying the same 3D model (**middle**), a screenshot from an external video filming the real scene and the two phones of the visitors (**right**).

Another very important use case was to run the application with multiple users in the same augmented space. Figure 12 showcases this scenario, where a real space was augmented with a 3D virtual model. Two different smartphones, a Google Pixel 2 and a Google Pixel 3, running the CultReal solution, display the same 3D model according to their position and orientation, as computed by the computer vision-based localization component.

## 6. Discussions and Conclusions

Beacon-based localization, the augmented images functionality, and the computer vision-based localization were tested both individually and combined in various scenarios. The pilot implementations were successful, but they uncovered several limitations that should be addressed before starting a more elaborated user testing process.

One problem was that the Bluetooth communication between the smartphone and the beacons was occasionally unstable. Also, the readings of the distances between the beacons and the visitor’s smartphone were sometimes delayed. This poses a problem when having multiple beacons in a cultural space, since the most proximal beacon is not recognized instantly as the visitor moves, introducing a lag in downloading the multimedia content and the annotated images from the CMS, which are necessary for the computer vision-based localization component. To solve this, we intend to acquire additional beacons from various manufacturers, and to select the ones that offer stable and fast communication with the smartphones. Also, we plan to start researching and debugging the Bluetooth connection stability in order to identify if the current problems are the result of the protocol, limitations of the hardware, or the speed of movement.

Another observed limitation was the difference between environments when configuring the space and when navigating as a visitor. These differences may be caused by the lighting conditions, shadows, camera angle, or by changes in the setting that may be introduced, for example, by rearranging the furniture. Lighting differences may influence the image matching algorithms, thus having a negative impact on the computer vision-based localization method. One possible solution is to provide multiple configurations of the same room by acquiring images annotated with position and orientation data at different moments of the day, in various lighting conditions. If there are major changes in the placement of objects in a room, the location configuration step must be redone.

During the pilot implementations, possible issues regarding the configuration of the locations and the placement of virtual elements in a scene were also observed. The location manager should pay attention when defining the coordinate system associated with a room and should acquire sufficient images of the environment for accurate image matching and position estimation based on computer vision. For an intuitive placement of the 3D multimedia objects in space, the origin and axes of a room’s coordinate system should be carefully chosen. When configuring a small room, a handy solution is to choose the origin as one of the corners of the room, with the axes defined along the frontiers between two adjacent walls or between a wall and the floor. However, when configuring a section of a big hall, the lack of landmarks might become an impediment. Momentarily, the placement of a virtual object in the scene is done by configuring its translation, rotation, and scale factors relative to the room’s coordinate system, without having a 3D visualization of the elements from the real environment. In the future, we intend to obtain a 3D reconstruction of the scene and to place it in the same virtual space as the multimedia objects, relative to the user-defined coordinate system. The visualization of the 3D reconstruction of the environment will provide location managers with a more intuitive method of placing the multimedia objects relative to the 3D model of the scene, regardless of the origin and axes of the room’s coordinate system.

The solution was piloted in four locations with various cultural purposes: an engineering museum, a library, a village museum, and a theatre. We tested the ease of use from the point of view of a location manager, as well as the intuitiveness and the response time from the visitor’s perspective. The fused localization, based on beacons and computer vision, provided a fast and accurate estimation of the position and orientation of the visitor’s device, and the integration of the ARCore functionalities (the Augmented Images API, the ARCore localization) in the application provided an intuitive user experience.

The configuration of each location, consisting of the scanning of the rooms and sections of interest and the placement of the multimedia objects, took several hours (from 4 to 8 h), depending on the number of areas of interest and on the number or characteristics of the virtual objects. This result is quite promising, demonstrating the potential of the fast deployment of the CultReal solution in any new cultural space.

## Figures and Tables

**Table 1:** Comparison between the ground truth (represented by the *VC_b_gt_* camera) and the positions and orientations computed with PnP (*VC_b_* camera).

Entry	Camera	Position (Meters)			Orientation (Quaternions)				Position Error (Meters)	Orientation Error (Degrees)
		*x*	*y*	*z*	*x*	*y*	*z*	*w*		
1	*VC_b_gt_*	6.66	−1.31	4.8	−0.0563	−0.3555	−0.1061	0.9269	-	-
	*VC_b_*	6.6919	−1.3123	4.7744	−0.0559	−0.3556	−0.1061	0.9268	0.0409	0.0001
2	*VC_b_gt_*	7.3939	−2.6273	0.6442	−0.1546	0.9357	0.176	−0.263		
	*VC_b_*	7.4062	−2.6357	0.6588	−0.1547	0.9356	0.1766	−0.263	0.0208	0.0001
3	*VC_b_gt_*	−5.5686	0.66349	2.3916	0.2158	−0.954	0.0632	−0.198		
	*VC_b_*	−5.5721	0.6647	2.4197	−0.2157	0.9541	−0.0633	0.197	0.0283	0.0685
4	*VC_b_gt_*	0.7498	−1.0597	−6.2537	0.0731	0.4312	0.1274	−0.8902		
	*VC_b_*	0.7784	−1.0615	−6.2734	−0.0726	−0.4315	−0.1274	0.8901	0.0347	0.0884
5	*VC_b_gt_*	−10.882	0.9963	−7.3946	0.0496	−0.3465	0.0976	−0.931		
	*VC_b_*	−10.893	1.0065	−7.4233	−0.0486	0.3461	−0.0973	0.931	0.0323	0.1186
μ	*VC_b_*	-	-	-	-	-	-	-	0.0314	0.0551

**Table 2:** Differences between ground truth, provided by ARCore, and estimated positions and orientations, obtained by running the PnP algorithm with FLANN, respectively with BFMatcher, with and without filtering.

Entry	Localization Method	Position (Meters)			Orientation (Quaternions)				Position Error (Meters)	Orientation Error (Degrees)
		*x*	*y*	*z*	*x*	*y*	*z*	*w*		
1	ARCore	−0.1	−1	−0.8	−0.1	−0.1	0	1	-	-
	PnP + FLANN	−0.08	−1	−0.79	0.05	−0.14	−0.01	0.98	0.01	10.15
	PnP + BFMatcher	−0.1	−1	−0.8	0.06	−0.14	−0.01	0.98	0	11.21
	PnP + BFMatcher + filter	−0.08	−1.01	−0.78	0.06	−0.14	−0.01	0.98	0.02	11.21
2	ARCore	1	1	2.8	0.9	0	−0.4	0	-	-
	PnP + FLANN	1.04	1.02	2.76	−0.88	0	0.4	−0.01	0.06	21.37
	PnP + BFMatcher	1	1	2.8	−0.89	0	0.44	−0.01	0	20.54
	PnP + BFMatcher + filter	1	1	2.81	−0.89	0	0.44	−0.01	0.03	20.54
3	ARCore	0.1	−1	−0.1	−0.2	−0.2	0	1	-	-
	PnP + FLANN	0.13	−1	−0.18	0.16	−0.26	−0.01	0.95	0.08	27.27
	PnP + BFMatcher	0.1	−1	−0.1	0.15	−0.26	−0.01	0.95	0	26.68
	PnP + BFMatcher + filter	0.1	−0.99	−0.11	0.15	−0.26	−0.01	0.95	0.01	26.68
4	ARCore	−0.3	0.1	−1	−0.1	0.4	0	0.9	-	-
	PnP + FLANN	2.96	−0.51	2.26	−0.7	0.66	0.07	−0.21	4.65	163.5
	PnP + BFMatcher	−0.3	0.1	−1	0.09	0.4	0	0.9	0	27.91
	PnP + BFMatcher + filter	−0.3	0.1	−1	0.09	0.4	0	0.9	0.08	27.91
5	ARCore	0.1	−0.9	−1	−0.2	0	0	1	-	-
	PnP + FLANN	0.08	−0.9	−1.01	0.18	0	0	0.98	0.01	38.18
	PnP + BFMatcher	0.1	−0.9	−1	0.17	0	−0.01	0.98	0	36.71
	PnP + BFMatcher + filter	0.12	−0.88	−1.05	0.17	0	−0.01	0.98	0.06	36.71
6	ARCore	0.2	0.9	0.8	1	0	−0.3	0	-	-
	PnP + FLANN	0.16	0.8	0.78	−0.98	0	0.18	−0.01	0.05	0.001
	PnP + BFMatcher	0.17	0.89	0.8	−0.98	0	0.19	0	0.02	0.001
	PnP + BFMatcher + filter	0.2	0.9	0.8	−0.98	0	0.19	0	0	0.001
7	ARCore	−0.4	−1.1	−0.9	−0.1	0.2	0	1	-	-
	PnP + FLANN	−0.42	−1.09	−0.88	0.11	0.19	0	0.97	0.03	0.001
	PnP + BFMatcher	−0.33	−1.12	−0.87	0.11	0.18	−0.01	0.97	0.07	0.001
	PnP + BFMatcher + filter	−0.4	−1.1	−0.9	0.11	0.18	−0.01	0.97	0	0.001
8	ARCore	−1.3	1	1.2	1	0	0.2	0.1	-	-
	PnP + FLANN	−1.38	1.19	1.31	−0.97	−0.01	−0.2	0.12	0.24	5.14
	PnP + BFMatcher	−1.37	1.19	1.22	−0.97	0	−0.2	0.12	0.21	6.86
	PnP + BFMatcher + filter	−1.3	1	1.2	−0.97	0	−0.2	0.12	0	6.86
9	ARCore	−0.5	−0.9	−2.6	−0.1	0	0	1	-	-
	PnP + FLANN	−0.4	−0.94	−2.66	0.08	−0.02	−0.02	0.99	0.12	18.1
	PnP + BFMatcher	−0.39	−1.04	−2.7	0.09	−0.01	−0.02	0.99	0.2	20.04
	PnP + BFMatcher + filter	−0.5	−0.9	−2.6	0.09	−0.01	−0.02	0.99	0	20.04
10	ARCore	−2	−1.1	0.2	−0.1	0.5	0.1	0.9	-	-
	PnP + FLANN	−2.22	−1.49	0.02	0.14	0.47	−0.07	0.86	0.48	11.9
	PnP + BFMatcher	−2.01	−1.1	0.18	0.08	0.49	−0.1	0.86	0.02	0.001
	PnP + BFMatcher + filter	−2	−1.1	0.2	0.08	0.49	−0.1	0.86	0	0.001
	PnP + FLANN	-	-	-	-	-	-	-	0.573	29.5612
μ	PnP + BFMatcher	-	-	-	-	-	-	-	0.052	14.995
	PnP + BFMatcher + filter	-	-	-	-	-	-	-	0.02	14.995

**Table 3:** The computing times of the feature matching algorithms, of the SIFT detector, of the selection of SIFT plus ARCore feature points and of the PnP algorithm. The number of SIFT key-points, the number of SIFT plus ARCore points and the final number of matched feature points used in the PnP algorithm, are also provided.

Entry	Feature Matching Method	SIFT Time (ms)	SIFT Key Points	SIFT + ARCore Time (ms)	SIFT + ARCore Points	Feature Matching Time (ms)	Number of Matched Points	PnP Time (ms)
1	FLANN	520.47	373	631.17	287	29.51	35	2.13
	BFMatcher					174.47	19	3.65
	BFMatcher + filter					237.07	19	0.85
2	FLANN	646.52	728	614.92	340	35.6	76	2.71
	BFMatcher					289.1	20	2.14
	BFMatcher + filter					531.11	20	0.92
3	FLANN	646.81	751	727.81	438	39	89	3.04
	BFMatcher					296.99	20	2.17
	BFMatcher + filter					588.48	20	9.01
4	FLANN	500	393	835.2	274	30.25	70	1.52
	BFMatcher					100.52	20	2.1
	BFMatcher + filter					208.15	20	0.97
5	FLANN	565.36	390	573.11	214	28.01	60	1.52
	BFMatcher					104.73	20	1.98
	BFMatcher + filter					147.52	20	0.88
6	FLANN	631.42	675	628.43	232	40	58	13.8
	BFMatcher					374.5	20	1.02
	BFMatcher + filter					197.75	20	2.02
7	FLANN	462.46	275	514.04	122	19.12	22	2.61
	BFMatcher					97.2	19	1.01
	BFMatcher + filter					27.47	19	2.53
8	FLANN	504.2	403	624.71	230	17.28	55	2.53
	BFMatcher					149.51	20	0.97
	BFMatcher + filter					77.48	20	2.24
9	FLANN	481.82	328	617.19	272	19.34	46	2.23
	BFMatcher					177.72	20	1.19
	BFMatcher + filter					92.73	20	2.43
10	FLANN	493.99	343	572.32	168	25.47	44	1.2
	BFMatcher					104.86	20	0.93
	BFMatcher + filter					69.03	20	2.06
	FLANN	545.305	466	633.89	258	28.358	56	3.329
μ	BFMatcher					186.96	20	1.716
	BFMatcher + filter					217.679	20	2.391

## Supplementary Materials

CultReal pilot in UPB Museum and UPB Library. Available online: https://youtu.be/Ep6--rfwtgg (accessed on 1 October 2021). CultReal pilot with multiple users. Available online: https://youtu.be/PIA59650RHs (accessed on 1 October 2021).
